# Supplementary material for: Differential expression of heat shock proteins and antioxidant enzymes in response to temperature, starvation, and parasitism in the Carob moth larvae, Ectomyelois ceratoniae (Lepidoptera: Pyralidae)
Source: PLoS One. 2020 Jan 29;15(1):e0228104. doi: 10.1371/journal.pone.0228104 (PMC6988935; doi:10.1371/journal.pone.0228104)
Supplement: S1 Fig — Insects were subjected to (A) heat: 46°C for one hour or (B) cold: -15°C for 30 min, and the percent dead was assessed. (PDF) [file pone.0228104.s001.pdf]

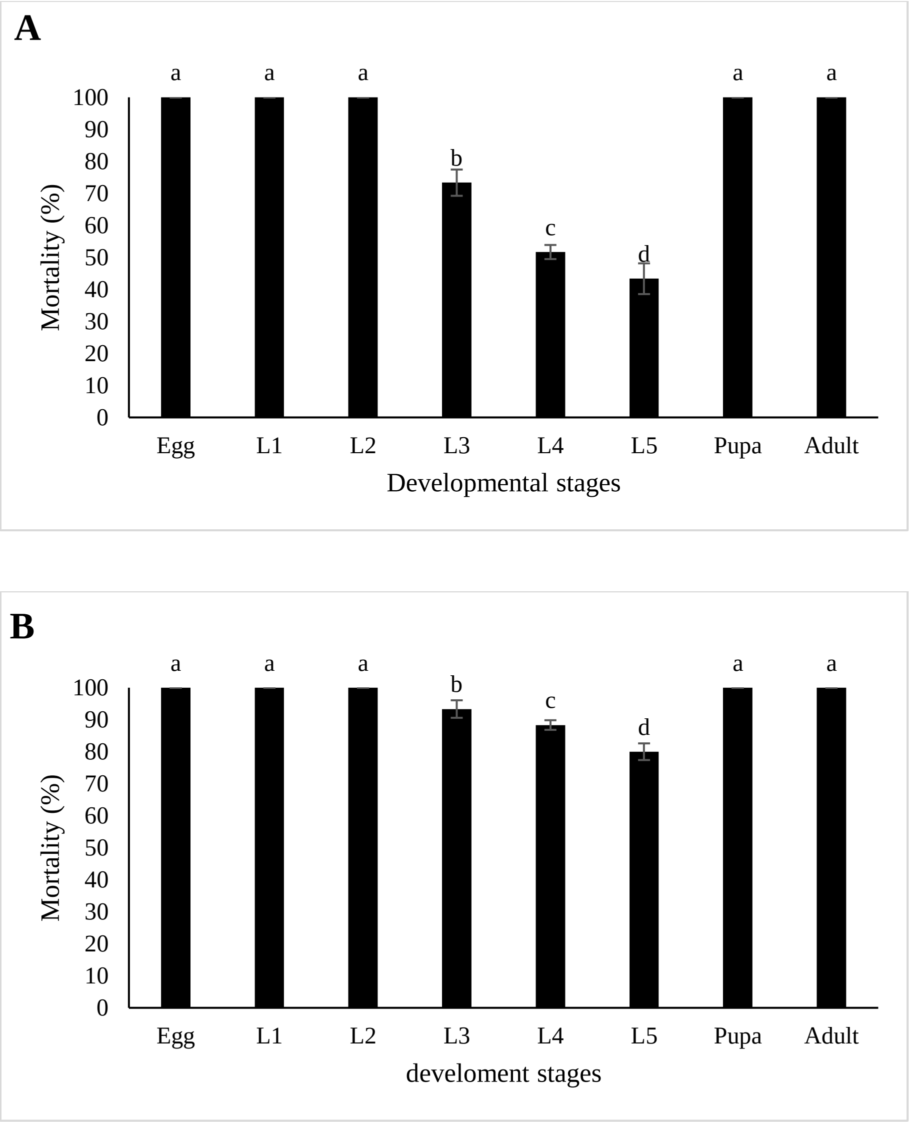


**Figure S1. Susceptibility of different developmental stages to extreme temperatures. Insects were subjected to (A) heat: 46°C for one hour or (B) cold: -15°C for 30 min, and the percent dead was assessed.**
