# Supplementary material for: Differential expression of heat shock proteins and antioxidant enzymes in response to temperature, starvation, and parasitism in the Carob moth larvae, Ectomyelois ceratoniae (Lepidoptera: Pyralidae)
Source: PLoS One. 2020 Jan 29;15(1):e0228104. doi: 10.1371/journal.pone.0228104 (PMC6988935; doi:10.1371/journal.pone.0228104)
Supplement: S2 Fig — Effect of exposure times on mortality of carob moth L5 larvae using (A) heat: 46°C for 120 min or (B) cold: -15°C for 30 min. (PDF) [file pone.0228104.s002.pdf]

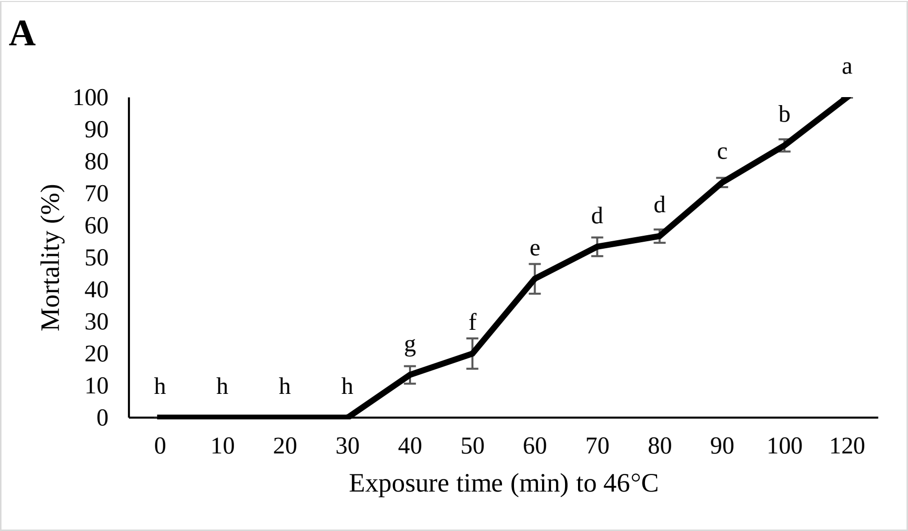

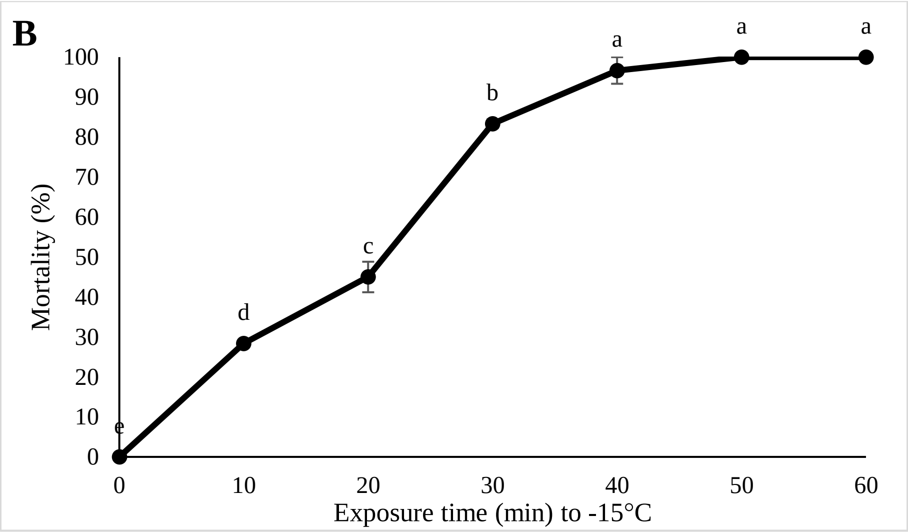

Figure S2. Effect of exposure times on mortality of carob moth L5 larvae using (A) heat: 46°C for 120 min or (B) cold: -15°C for 30 min.
