# Supplementary figures and images for: Differential expression of heat shock proteins and antioxidant enzymes in response to temperature, starvation, and parasitism in the Carob moth larvae, Ectomyelois ceratoniae (Lepidoptera: Pyralidae)
Source: PLoS One. 2020 Jan 29;15(1):e0228104. doi: 10.1371/journal.pone.0228104 (PMC6988935; doi:10.1371/journal.pone.0228104)

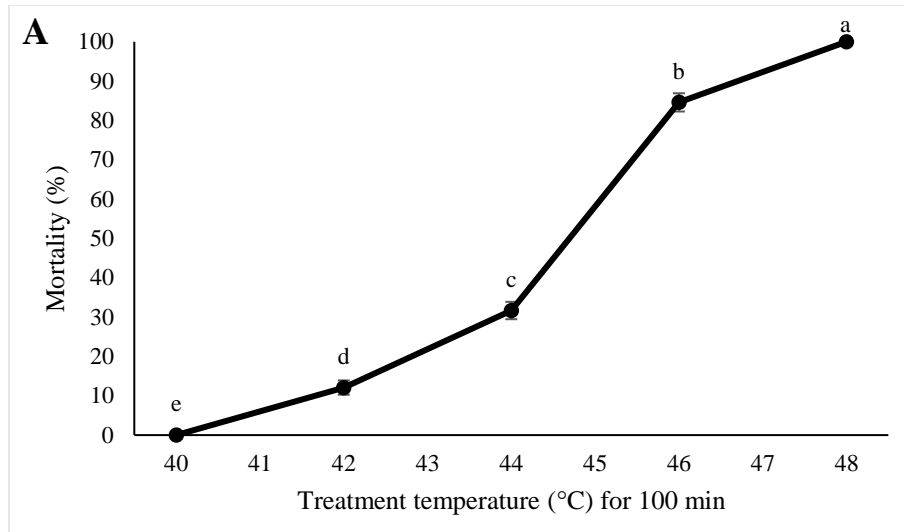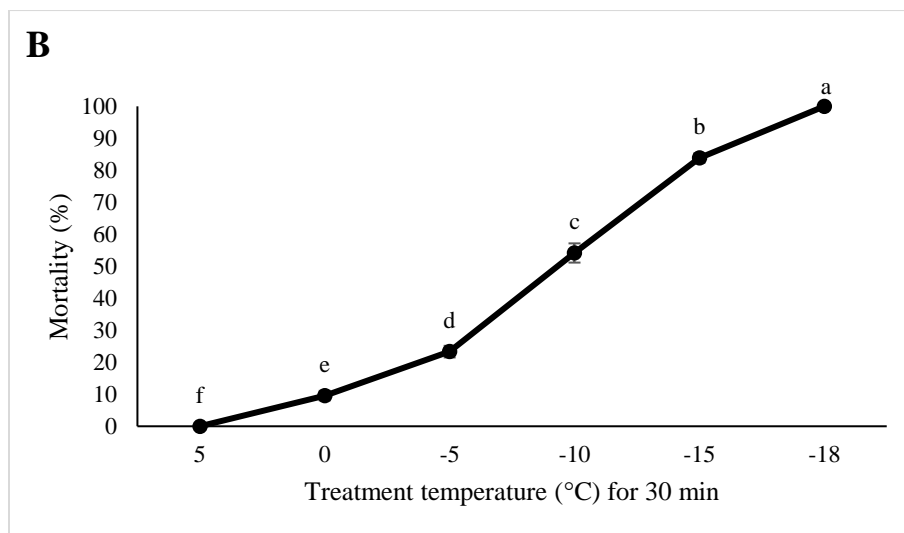

**Figure S3. Effect of different (A) high or (B) low temperatures on mortality of carob moth L5 larvae.**

Supplement: S3 Fig — Effect of different (A) high or (B) low temperatures on mortality of carob moth L5 larvae. (PDF) [file pone.0228104.s003.pdf]
