# Supplementary material for: Differential expression of heat shock proteins and antioxidant enzymes in response to temperature, starvation, and parasitism in the Carob moth larvae, Ectomyelois ceratoniae (Lepidoptera: Pyralidae)
Source: PLoS One. 2020 Jan 29;15(1):e0228104. doi: 10.1371/journal.pone.0228104 (PMC6988935; doi:10.1371/journal.pone.0228104)
Supplement: S5 Fig — Induction of extreme temperature tolerance by varying duration of (A) 37°C pretreatments or (B) 10°C pretreatments and then subjected to (A) 46°C for 100 min or (B) -15°C for 30 min. (PDF) [file pone.0228104.s005.pdf]

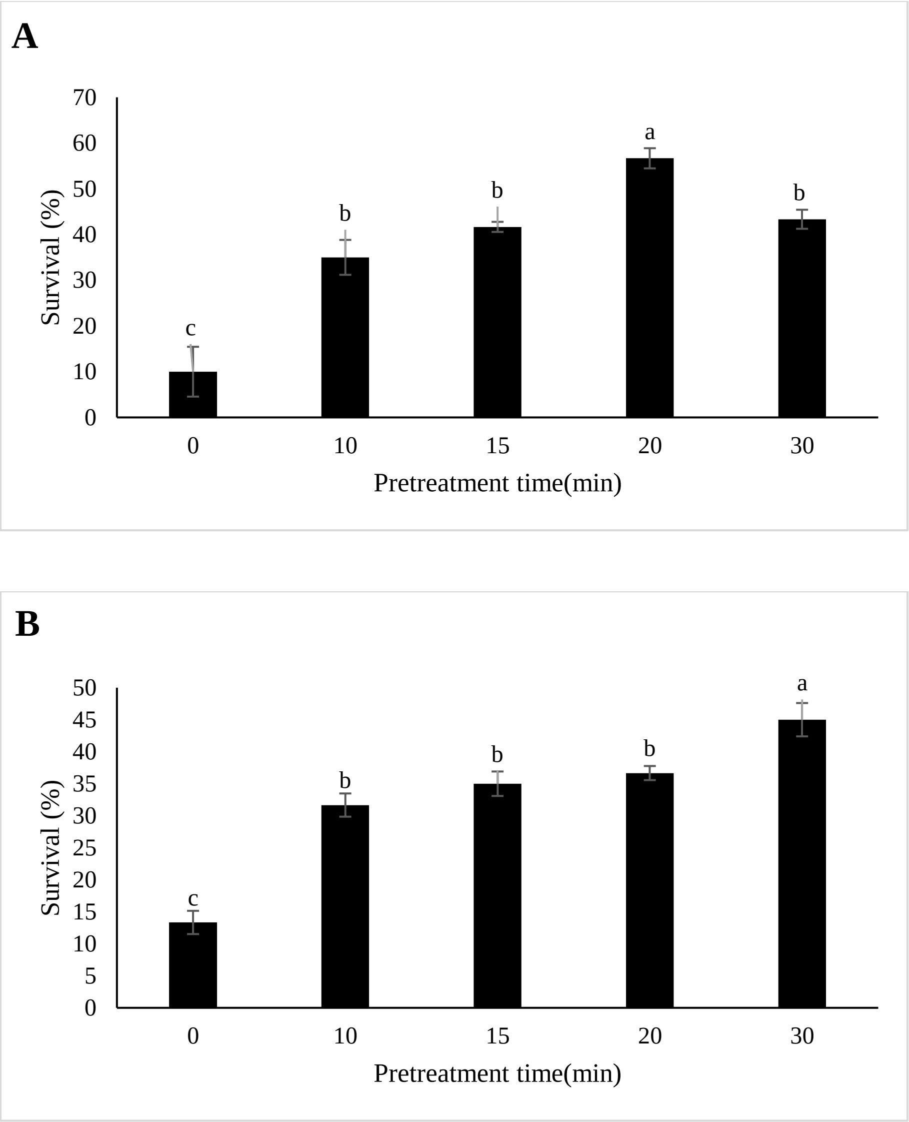

Figure S5. Induction of extreme temperature tolerance by varying duration of (A) 37°C pretreatments or (B) 10°C pretreatments and then subjected to (A) 46°C for 100 min or (B) -15°C for 30 min.
