# Supplementary material for: Differential expression of heat shock proteins and antioxidant enzymes in response to temperature, starvation, and parasitism in the Carob moth larvae, Ectomyelois ceratoniae (Lepidoptera: Pyralidae)
Source: PLoS One. 2020 Jan 29;15(1):e0228104. doi: 10.1371/journal.pone.0228104 (PMC6988935; doi:10.1371/journal.pone.0228104)
Supplement: Attachment — Submitted filename: Response to Reviewers.docx [file pone.0228104.s006.docx]

**Response to reviewers**

**Reviewer 1:**

1. **Comment one (line 39):**

Response:

Done.

2. **Comment two (line 61):**

Response:

Done.

3. **Comment three (line 106-107):**

Response:

We meant 18S ribosomal DNA (rDNA)-based primers. That was corrected in the manuscript.

4. **Comment four (line 124):**

Response:

The sequencing was performed commercially by Bioneer (Daejeon, Korea).

5. **Comment five (line 161):**

Response:

Individual larvae that did not move after touching with a small fine brush were counted as dead. Egg and pupal survivals were determined by hatch and adult emergence at 25 °C, respectively. That was added in the manuscript.

6. **Comment six (line 201):**

Response:

Done.

7. **Comment seven (line 203):**

Response:

We did not use any protease inhibitor during the homogenization for enzyme assays however, we tried to do our enzyme extraction at cold room and on ice as well.

8. **Comment eight (line 215):**

Response:

No, we did not use any antioxidant substances as did some other authors:

- Dubovskiy, I. M., Martemyanov, V. V., Vorontsova, Y. L., Rantala, M. J., Gryzanova, E. V., & Glupov, V. V. (2008). Effect of bacterial infection on antioxidant activity and lipid peroxidation in the midgut of Galleria mellonella L. larvae (Lepidoptera, Pyralidae). Comparative Biochemistry and Physiology Part C: Toxicology & Pharmacology, 148(1), 1-5.

9. **Comment nine (line 221): Line 221:**

Response:

Values were considered statistically different if p< 0.05. That was added in the manuscript.

10. **Comment ten (line 234):**

Response:

Done.

11. **Comment eleven (line 276):**

Response:

The term of suboptimal was changed to sublethal.

12. **Comment twelve (Figure 5):**

Response:

Insects were maintained at 26±1 °C in a growth chamber. For pre-treatment, animals were transferred to an incubator with 25 °C. Thus, there was no temperature variance during pre-treatment time.

13. **Comment thirteen (Figures 5 and 6):**

Response:

Data were ploted in figure 5 as you suggested. In figure 6 the value “0” shows that they did not subjected to any pre-treatment.

14. **Comment fourteen (Figures):**
    Response:

As you proposed we placed all data (Figures 2, 3, 4, and 5) in supplemental material as Figures S1, S2, S3, and S4.

15. **Comment fifteen (Figure 7):**

Response:

Correction was done as you suggested.

16. **Comment sixteen (Figures**

Response:

Correction was done.

**Reviewer 2:**

Reviewer #2: The goal of this MS is to understand the role of AOEs and HSPs in the Carob moth as it relates to temperature (low and high), starvation, and parasitism. The authors use a robust strategy that involves gene expression, biochemistry, survivorship and loss of function experiments. I think the results are interesting and worthy of publication.

My main concern with the MS has to do with background work and literature. The authors consistently cite new literature, in multiple cases papers that are less than five years old when older examples (10 to 15 yrs old) provide better background information and data. Specifically, the entire literate on heat treatments use in commodities handling is ignored; a growing area of research as the authors know. They also ignore, probably indirectly, a lot of the classic insect HSP and AOE work by Lee, Denlinger, and others. I was surprised and glad to see references to Sinclair’s HSP work but most of the author’s background comes from recent studies that are not very thorough. Additionally, the literature on temperature pre-treatments is also quite vast. It includes work on low temperature pretreatments like rapid cold hardening (also of Lee and Denlinger), rapid heat hardening, and hormesis. I mention this not to point out a flaw, but to suggest to the authors that the literature not being presented in here, might provide answers and mechanistic explanations that would strengthen their MS.

**Response**:

In a sense the reviewer is right and it we corrected as he mentioned.

My second main concern has to do with the tone of the MS. This is an insect MS written for an insect audience but sent to a general broad journal. The authors never explain the reason for their treatments. Why starvation? Why are those temperatures chosen? Why the lengths of treatment? And the parasitism? Does biological control of Ectomyelois involve parasitoids? The authors need to explain a lot about the reason behind their treatments and the biology of the moth in a non-entomology journal. I am an entomologist and work in a similar area, and I do not understand the point of starvation, in this context. Without explanations as to why certain treatments were chosen, the MS reads like a bunch of random experiments pasted together and I suspect that is not the case and that the treatments were chosen rather carefully. If this is a paper about “aoes and hsps in response to common non-pesticide control tactics for Carob moth” then fine, but please do tell us.

**Response:**

Starvation was done since we would like to know if there is any differential expressions exist between genes studied. Moreover, starvation is a stress that the insects encounter, particularly during large population outbreaks or during yeild harvest. As some studies have made a connection between starvation and stress responses, we wished to see if the same responses could be induced with this stress.

Temperatures were chosen based on literature review that shows these points usually used for Pyralid moth control in the storehouses (Kim et al. 2017). Also, these temperature and exposure lengths were selected to facilitate comparisons with similar studies.

Parasitoid wasps are being investigated for their potential to control carob moths, and hence, we were interesed in understanding if parasitism induced simialr stress responses in the insect. These sentences were added in the MS.

1. **Comment one (line 53):** Line 53, the appropriate reference about insect antioxidant systems is not paper from 2015. I believe there are multiple papers in the 1990s (archives of insect biochemistry had an entire antioxidant issue in 1997, I believe), and dozens of other papers between then and 2015.

Response:

Reference was added (Felton & Summers,1995).

2. **Comment two (line 57):**

Response:

The reference for insect non-enzymatic antioxidant was added.

3. **Comment three (line 60):**

Response:

As the reviewer mentioned our emphasize was on those stressors which were related to our studies. However, those points that reviewer mentioned were added to the MS.

4. **Comment four (line 64):**

Response:

Done.

5. **Comment five (line 65):**

Response:

Done.

6. **Comment six (line 162):**

Insects live and grow in a limited range of temperatures, but suffer significant mortality in extreme temperature. So heat and cold temperatures have been used to control stored- product insects because they are relatively inexpensive and highly effective. In the large scale of application, heat and cold treatments may take some time to reach a lethal high/low temperatures and may induce an acclimation of insect larvae to high/low temperatures in the process and may lead to a significant reduction of control efficacy. So we tried to measure the effects of sublethal temperatures during different times on control. Otherwise, we chose mentioned temperatures and exposure times based on preliminary screening before they have been chosen. These sentences were added in the MS.

7. **Comment seven (line 177):**

**Response:**

Organisms are able to survive exposure to high/low temperature levels by an acclimation (25-26), which is a process that associated with the production of HSPs, antioxidant enzymes/compounds, polyols and sugars. Short-term acclimation and/or long-term evolutionary adaptation are process that insects can alter their sensitivity to heat/cold stress (27). Short-term heat/cold acclimation enabling insect to adapt to variable environmental temperatures (28- 31). Rapid cold hardening with acclimation for 2 h at 4ºC of recently-eclosed adults of five coleopteran species associated with stored grain significantly increased the survival time at various sub-zero temperature compared to those without acclimation (32). Yocum and Denlinger (19) showed that a mild heat treatment of 40°C for 2 h to the flesh fly, *Sarcophaga crassipalpis*, conferred thermal tolerance to a subsequent normally lethal heat treatment of 90 min at 45°C.

8. **Comment eight (line 190):**

Response:

Number of replication for gene expression and enzyme assays were three times, and each assay in triplicate. Number of RNAi bioassay replication was six times and each time include 80 larvae with 10 larvae in each Petri dish (diameter of 9 cm). Number of replication for starvation was 8 replications and each replication included 10 larvae in each Petri dish. (diameter of 9 cm).

9. **Comment nine (line 253):**

Response:

In temperature tolerance bioassays, larval mortality was recorded after two-hour recovery periods at 25°C. Egg and pupal survivals were determined by hatch and adult emergence at 25 °C, respectively.

10. **Comment ten (line 344):**

Done.

11. **Comment eleven (line 360):**

Done.

12. **Comment twelve (line 394):** Line 394, “the insects responded accordingly by increasing both SOD and CAT…” They did, however lipid peroxidation damage was detected anyway. Is this really a protective release or a curative one? The authors make it sound like the insects are protecting against oxidative damage, but ox damage already happened. The statement needs revision.

Response:

The insects responded to this stress by increasing both SOD and CAT to manage the increased levels of ROS compounds. In conjunction with these curative antioxidant responses, the stress treatments also increased expression of one or both of the two HSPs examined in this study.

13. **Comment thirteen (line 410):**

Response:

In the carob moth, injection of dsRNA into the hemocoel proved effective in reducing the targeted genes’ transcripts, demonstrating that this lepidopteran species is somewhat amenable to this useful molecular biology tool. It is worth noting however, knockdown of HSP90 (25% at 24 h post-injection) was considerably weaker than that of HSP70 (90% at 24 h post-injection), and the knockdown was only temporary for HSP90. Similarly weak and transient RNAi-mediated knockdowns are common in many lepidopteran species (45, 46), but in the carob moth, the knockdown was nevertheless sufficient to confirm that HSP90 plays an important role in conferring thermotolerance.

14. **Comment fourteen (line 417):**

Response:

The differential increases of the two HSPs’ transcripts with variable stresses reflects differences in both their inducibility with different stresses as well as their constitutive levels of expression prior to a stress. While both HSPs are capable of acting as molecular chaperones to assist in protein folding under non-stressed conditions, they do have different roles that may reflect different induction rates with different stresses
